# Using EpiCore to Enable Rapid Verification of Potential Health Threats: Illustrated Use Cases and Summary Statistics

**DOI:** 10.2196/52093

**Published:** 2024-03-15

**Authors:** Nomita Divi, Jaś Mantero, Marlo Libel, Onicio Leal Neto, Marinanicole Schultheiss, Kara Sewalk, John Brownstein, Mark Smolinski

**Affiliations:** 1 Ending Pandemics San Francisco, CA United States; 2 Department of Epidemiology and Biostatistics Mel and Enid Zuckerman College of Public Health University of Arizona Tucson, AZ United States; 3 Computational Epidemiology Lab Boston Children’s Hospital Boston, MA United States; 4 Harvard Medical School Harvard University Boston, MA United States

**Keywords:** disease surveillance, surveillance, verification, early detection, epidemic intelligence, risk assessment, threat, threats, crisis, crises, outbreak, outbreaks, warning, warnings, crowdsource, crowdsourcing, surveillance, digital health, detect, detection, risk, risks

## Abstract

**Background:**

The proliferation of digital disease-detection systems has led to an increase in earlier warning signals, which subsequently have resulted in swifter responses to emerging threats. Such highly sensitive systems can also produce weak signals needing additional information for action. The delays in the response to a genuine health threat are often due to the time it takes to verify a health event. It was the delay in outbreak verification that was the main impetus for creating EpiCore.

**Objective:**

This paper describes the potential of crowdsourcing information through EpiCore, a network of voluntary human, animal, and environmental health professionals supporting the verification of early warning signals of potential outbreaks and informing risk assessments by monitoring ongoing threats.

**Methods:**

This paper uses summary statistics to assess whether EpiCore is meeting its goal to accelerate the time to verification of identified potential health events for epidemic and pandemic intelligence purposes from around the world. Data from the EpiCore platform from January 2018 to December 2022 were analyzed to capture request for information response rates and verification rates. Illustrated use cases are provided to describe how EpiCore members provide information to facilitate the verification of early warning signals of potential outbreaks and for the monitoring and risk assessment of ongoing threats through EpiCore and its utilities.

**Results:**

Since its launch in 2016, EpiCore network membership grew to over 3300 individuals during the first 2 years, consisting of professionals in human, animal, and environmental health, spanning 161 countries. The overall EpiCore response rate to requests for information increased by year between 2018 and 2022 from 65.4% to 68.8% with an initial response typically received within 24 hours (in 2022, 94% of responded requests received a first contribution within 24 h). Five illustrated use cases highlight the various uses of EpiCore.

**Conclusions:**

As the global demand for data to facilitate disease prevention and control continues to grow, it will be crucial for traditional and nontraditional methods of disease surveillance to work together to ensure health threats are captured earlier. EpiCore is an innovative approach that can support health authorities in decision-making when used complementarily with official early detection and verification systems. EpiCore can shorten the time to verification by confirming early detection signals, informing risk-assessment activities, and monitoring ongoing events.

## Introduction

### Background

World Health Organization (WHO) leadership has recently suggested the need for collaborative surveillance within and beyond the health sector for more robust event detection, risk assessment, and response monitoring [1-3]. Furthermore, the recent quadripartite partnership among the Food and Agriculture Organization of the United Nations, the World Organization for Animal Health, the UN Environment Programme, and the WHO has noted the need for intelligence systems to use an integrated “One Health” approach to reduce the risk of ongoing and emerging threats [2].

Although early warning “signals” from such systems are leading to earlier detection and swifter responses to emerging threats, the proliferation of these systems can also generate a large volume of data that must be processed before contributing toward the early warning or risk assessment of ongoing threats [3]. This large volume of data may also result in false alarms that might propagate rumors and quickly overwhelm the surveillance infrastructure [2,4]. This risk of false alarms underscores the crucial role of human input for data curation and signal verification.

Delays in the effective response to a genuine health threat are often the result of the time to verification of a health event—determining if an unofficial reported threat is real so an appropriate response can ensue. An analysis by Chan et al [5] based on WHO-verified outbreaks reported in Disease Outbreak News noted median time from “outbreak start” to “outbreak discovery” dropped from 29.5 days (95% CI 13-59 days) to 13.5 days (95% CI 3.5-44.5 days) during that time. More recently, the time to detection has been reduced due in part to the advancement of digital epidemiology, but the task of timely verification remains a challenge [5,6]. WHO has presented data on additional delays in verifying that outbreaks are indeed real events, noting up to a 1-week delay [7]. It was the extra several days for outbreak verification that was the main impetus for creating EpiCore, a virtual multi-sectoral community of human, animal, and environmental health professionals that supports the rapid verification of potential health threats. EpiCore aims to contribute to the reduction in time to verify human, animal, or environmental health events to obtain event details within 24 hours of initiating a request for information (RFI) within a digital secure information system. The objective of this paper is to describe the potential of the crowdsourcing of information from qualified professionals through EpiCore to facilitate the verification of early warning signals of potential outbreaks and support the monitoring and risk assessment of ongoing threats.

### About EpiCore

Conceptualized in 2012 by Ending Pandemics as an initial partnership between the TEPHINET (Training Programs in Epidemiology and Public Health Interventions Network) [8,9], Program for Monitoring Emerging Diseases (ProMED)–mail [10], and HealthMap of Boston Children’s Hospital, EpiCore became operational in 2016 [11]. EpiCore does not aim to replace any official verification system and is meant to be a complementary verification tool. An epidemic intelligence expert in the role of the focal point oversees the system’s operations. This individual is responsible for ensuring the system is accessible and running 24/7, providing support and training to the users of the system and ensuring privacy protocols are being followed. The focal point also oversees communications between the various users as well as the distribution of a quarterly newsletter.

Several epidemic intelligence and disease prevention and control organizations use EpiCore to confirm signals detected from primarily digital sources. These groups are referred to as “requesters” in the system. Requesters have included members from ProMED, GeoSentinel, HealthMap, the Hungarian National Association of Radio Distress-Signaling and Info-communication that operates the Emergency and Disaster Information Services, and Médecins Sans Frontières (MSF) Operational Centre Barcelona [10,12-15]. Requesters send out RFIs, a specific question sent through EpiCore about known or potential “events” to EpiCore responders (see below) for verification.

The virtual community of health professionals in EpiCore are referred to as “responders” in the system. To become an EpiCore responder, a health professional applies and must have *at least two* of the following qualifications: (1) a degree in public health (eg, MS, MPH, and PhD) or a related field (eg, MD, DVM, and RN); (2) health profession certification or licensure (eg, livestock officer and food inspector); (3) at least 3 years of experience in human or animal health or environmental health; (4) affiliation with a medical center, ministries or departments of health, or other health-related organizations (eg, nongovernmental organizations and private sector); and (5) successful completion of a field epidemiology training program. In the first 5 years after it became operational in 2016, EpiCore membership grew to over 3300 individuals with members spanning 161 countries. Over one-third of members identify themselves as experts spanning multiple sectors (n=1134, 34.3%). Most members (n=2807, 84.9%) list themselves as human health experts; 926 (28%) listed themselves as animal health experts, and 962 (29.1%) as environmental health experts. Responders join the network and contribute as *individuals* (ie, they do not represent any organization and their contributions can be provided anonymously if so desired). As an incentive to increase participation, responders have access to select resources related to infectious disease prevention and control, including web-based courses and publications.

**Figure 1:**
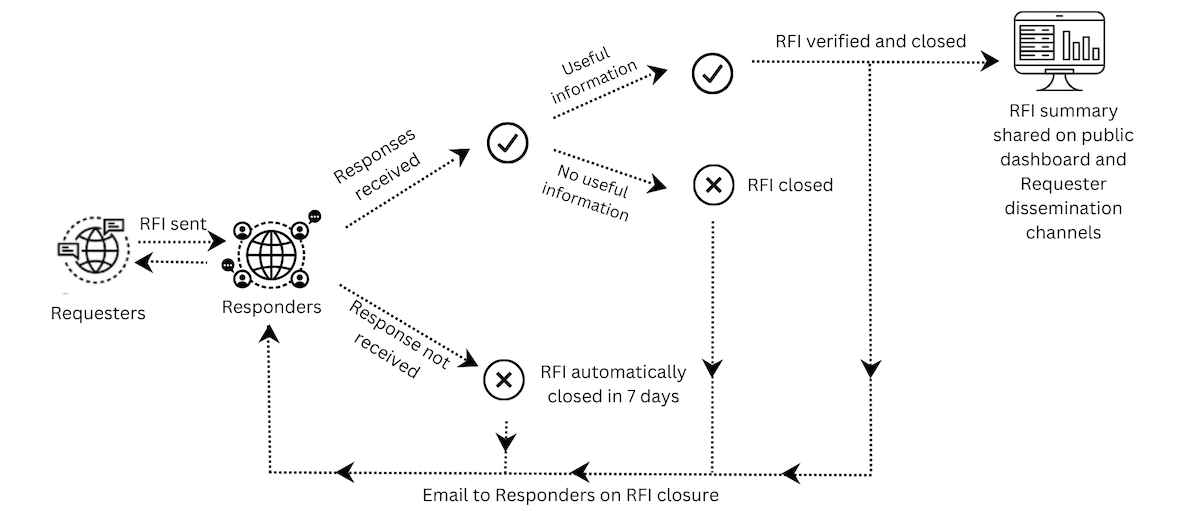
Illustration of key steps in the EpiCore verification process. RFI: request for information.

When evidence of a health event is detected, local information is requested from EpiCore responders to rapidly provide details on the event (Figure 1). Responders near (in the district, country, or region) where events are reported are selected, in essence, providing the “eyes and ears” on the ground. The radius around the event where responders are selected depends on the density of the responders in the location of the potential event. In areas with fewer responders, the radius is increased by requesters to the country or regional level to include more responders. Through EpiCore’s secure digital platform, responders with knowledge of the event can easily and quickly provide details and expert opinions to inform event verification or ongoing event monitoring. Information provided by responders can only be seen by requesters. Other EpiCore responders are unable to see individual responses to ensure the privacy and anonymity of the responders. However, all EpiCore requesters can view the RFIs from other requesters as well as the responses to those requests. This ensures that information silos between requesters and their organizations are minimized.

An RFI is considered verified if an EpiCore requester deems sufficient reliable additional details have been collected from the responders to confirm a reported event is true, or enough details have been collected to confirm the reported event or early warning signal is a false alarm. RFIs are considered verified if the report is confirmed to be valid or a false alarm. EpiCore requesters give the highest priority to reliable sources of information, including official statements and complementary reports or documents obtained at the locality of the reported event. Requesters create an RFI summary of verified health events, which constitutes a synopsis of the information received via the platform as well as other information publicly available that contributes to the understanding of the situation. These RFI summaries are made available on EpiCore’s public dashboard as well as via the requesters’ own dissemination channels, so anyone can have access to details on events verified by EpiCore [11,16]. Requesters are urged to close RFIs as soon as helpful information is available to ensure timely dissemination of the information to the public. If no information is received from responders, RFIs are automatically closed after 7 days.

In addition to disseminating information via the public dashboard, EpiCore is also contributing information to the Epidemic Intelligence from Open Sources platform and supports the goal of the WHO Hub for Pandemic and Epidemic Intelligence to combine information from traditional surveillance, event-based surveillance, participatory or community surveillance, and on-the-ground investigations with contextual information to generate an assessment of public health risk [17]. EpiCore is also facilitating organizations to improve their information-dissemination activities. For example, ProMED noted an increased number of responses to their RFIs about health events on the EpiCore platform compared to requests sent through their network [10,11].

## Methods

### Study Design

Data on the EpiCore platform from January 2018 to December 2022 were analyzed to evaluate key descriptive statistics, as well as to identify use cases illustrative of the various utilities of the system. All RFIs created over the 5 years were analyzed to calculate the annual mean and median time to reply to an RFI. Annual RFI response rates and verification rates were also calculated. RFIs that received a response, regardless of whether the information was considered useful or not, were categorized as “RFIs with a response.” The response rate was calculated as the number of RFIs with a response as a percentage of the total number of RFIs to indicate members’ engagement on the platform. All RFIs that requesters deem to have sufficient and useful information through responder contributions with additional information to confirm an event or to debunk misinformation or disinformation were categorized as “verified.” The verification rate was calculated as the number of RFIs that the requesters have classified as verified as a percentage of the total number of RFIs with a response. This rate informs the proportion of RFIs obtaining information from EpiCore members that were deemed useful in the verification of a reported event.

### Ethical Considerations

This study does not constitute human subject research as it is a descriptive analysis of a system. The data collected were limited to the time of information flow and summarization of information about health events to describe EpiCore. The data set has no personal identifiers. This rationale is consistent with the Harvard University policies on human subjects research [18].

## Results

### EpiCore Platform and Statistics

During the observed period, 622 RFIs were sent out globally. Of these RFIs, 485 (78%) were related to health events regarding human exposure, 121 (19.4%) to health events regarding animal exposure, 12 (1.9%) to health events with human and animal exposure, and 4 (0.6%) to environmental health events. A total of 398 RFIs received a response during the observed period. The mean and median response times are indicated in Table 1.

**Table 1:** EpiCore key summary statistics on all RFIs^a^ issued by year between 2018 and 2022.

Time period	Total RFIs sent, n	RFIs with a response, n	Response time (h), mean (SD)	Response time (h), median (IQR)	Response rate (%)	Reacted within 24 h, n (%)
2018	205	134	19.5 (40.6)	4.2 (16.5)	65.4	108 (80.6)
2019	167	100	17.1 (35.1)	5.2 (15.8)	59.9	81 (81)
2020	96	61	12.1 (26.24)	2.2 (6.9)	63.5	54 (88.5)
2021	106	70	17.4 (31.6)	4.2 (14.3)	66	57 (82.4)
2022	48	33	10.1 (26.9)	1.6 (6.2)	68.8	31 (94.0)

^a^RFI: request for information.

**Figure 2:**
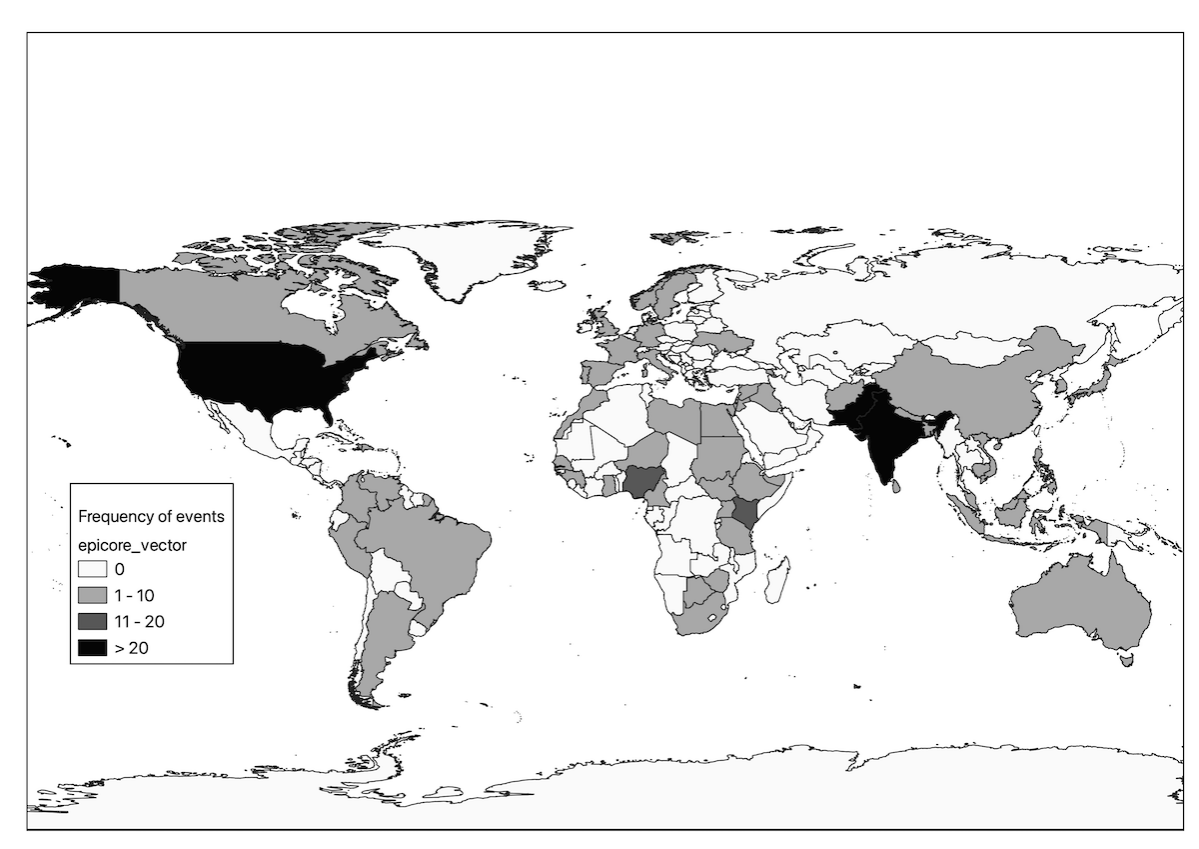
Map of frequency of EpiCore RFIs by location of potential event between 2018 and 2022. RFIs represented in this map are generated by EpiCore requesters for additional input for verification by EpiCore responders. The map is not a representation of all potential health events during the observed period, but is limited to the subset of events identified by the requesters on EpiCore. RFI: request for information.

Figure 2 illustrates the locations of the RFIs with a response, demonstrating the geographic distribution of the RFIs over the 5 years. A majority of the RFIs with a response received responses within 24 hours (n=334, 84%). Furthermore, 247 (62.1%) RFIs of those that received a response (n=398) were deemed to have sufficient and useful information through responder contributions to verify the reported event and were summarized on the public dashboard. The annual verification rate has increased each year from 2018 to 2022 ranging from 50.7% to 84.8% (Table 2). The response rate increased from 65.4% in 2018 to 68.8% in 2022.

**Table 2:** EpiCore key statistics on all RFIs^a^ with a response by year between 2018 and 2022.

Time period	RFIs with a response, n	RFIs verified, n	Verification rate (%)
2018	134	68	50.7
2019	100	57	57
2020	61	37	60.7
2021	70	57	81.4
2022	33	28	84.8

^a^RFI: request for information.

### EpiCore Illustrative Use Cases

EpiCore plays a crucial role in reducing the time to verification by confirming early detection signals, informing risk assessment activities, and monitoring ongoing events. Gathering timely information is pertinent both during the early days of a potential outbreak and for continued situational awareness. EpiCore provides access to a network of health experts who can share information about a specific event in response to an RFI. Information provided by members of this trusted network is valuable, including knowing how a disease is spreading within a country, if an outbreak has crossed borders, the epidemiological characteristics, severity and transmissibility, and laboratory results. The following case studies highlight the various uses of EpiCore.

### Case Study 1: Ruling Out False Alarms

Unnecessary alarms can result in wasted valuable resources in responding to essentially a nonevent. EpiCore members provide information to help rule out false alarms that may circulate through various media outlets, especially during the early days of a potential infectious disease outbreak. For example, EpiCore members provided timely information during an uptick in rash illness observed in November 2021 among fishermen in Senegal as reported in the local media. EpiCore responders provided information within 24 hours of the RFI being sent to those within the geographical area of the reported “event.” Their responses clarified that the health conditions were likely caused by environmental factors and not an infectious disease [19].

### Case Study 2: Real Time Details on an Evolving Event

In another situation in October 2020, local media in South Sudan reported 3 sudden deaths in Western Bahr el Ghazal State in patients presenting with hemorrhagic symptoms, raising fears of Ebola. Responders confirmed the occurrence of the event and shared additional specifics that also quoted national health authorities. They noted that the 3 reported cases were buried before the arrival of the rapid response team and samples could not be taken for further laboratory investigation. It was also shared, however, that 3 more cases presenting with similar symptoms were still alive in the same area and tested negative for Ebola and other hemorrhagic fever viruses. The RFI was summarized, documenting the lack of laboratory results for the first cases and reporting that additional cases tested negative for hemorrhagic fever viruses, hence quelling the fears of Ebola [20].

### Case Study 3: Tracking an Unknown Pathogen

**Figure 3:**
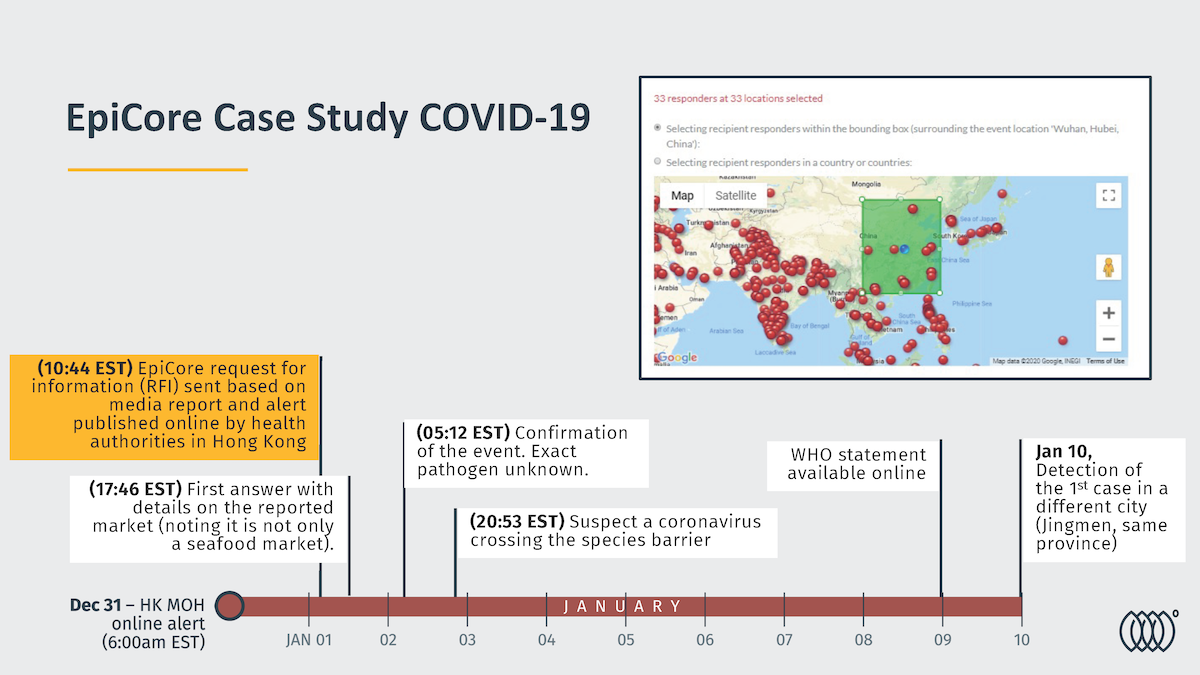
Timeline of information provided on EpiCore along with other key releases of information from authorities during the early days of the COVID-19 pandemic. Dec: December; HK: Hong Kong; Jan: January; MOH: Ministry of Health; RFI: request for information; WHO: World Health Organization.

In an event that garnered the world’s attention, local media reported on December 31, 2019, that health authorities in Wuhan (Hubei, China) were investigating an unexplained cluster of severe respiratory disease that had sickened at least 27 people within a short period. A viral pathogen was suspected, raising rumors of severe acute respiratory syndrome on several media outlets. It was also suggested that some cases were linked to a seafood market in Wuhan city. The same cluster of an unknown illness was noted in a web-based report by Hong Kong’s Centre for Health Protection (December 31, 2019). EpiCore responders in proximity to Wuhan (n=36) were sent an RFI to provide any additional details to document the reported event and provide information on the level of local spread. Initial responses were provided within 7 hours, confirming the event’s occurrence. Figure 3 illustrates the timeline of key responses received for this RFI. Among the received responses, 1 responder reported that the seafood market in Wuhan also sold wild animals, noting that the risks of zoonotic disease were significant. On January 2, 2020, responders mentioned that local authorities suspected that the responsible pathogen was likely a known coronavirus able to cross species (spillover). In the following days, the network noted that around 60 pneumonia cases had been identified at Jinyintan Hospital in Wuhan. The network also provided preliminary information on at least 15 laboratory-diagnosed cases with a new strain of coronavirus (influenza, avian influenza, adenovirus, SARS-CoV-1, and MERS [Middle East Respiratory Syndrome] were ruled out). On January 10, members shared information about the first occurrence of cases in a different city (Jingmen, the same province as Wuhan). In addition, information was shared about the first cases with no known or substantiated exposure to the Wuhan market. Responders also noted a particular concern about the proximity of this outbreak to the Chinese New Year with the vast exodus of people from Wuhan to rural areas to come in the following weeks. The RFI was summarized for the public dashboard and further shared on EpiCore’s first quarterly newsletter in 2020 [21,22].

### Case Study 4: Supporting Decision-Making

In August 2018, MSF Operational Center in Spain [23] received a report about several human deaths and an illness from an unknown disease in Douna, Mali. All cases experienced an extreme burning or heating feeling in the legs with edema often followed by “blackening” of the skin. MSF issued an RFI on EpiCore to obtain details to help with their risk assessment. Responders confirmed the occurrence of this event, which had been observed for several months. They also shared the investigation report from local health authorities that indicated that the cases had likely had acute severe malnutrition (scurvy and beriberi) due to a significant food shortage in the affected communities. MSF used the information provided by the responders for risk assessment purposes [24].

### Case Study 5: New and Evolving Presentation of Disease

EpiCore has also proven pertinent for monitoring diseases involving multiple countries. In May 2021 for example, the incidence of a rare fungal infection, mucormycosis, was observed to be increasing across India in patients with COVID-19; EpiCore responders from countries with historically reported mucormycosis were asked to provide their professional opinion and share clinical details observed in their own countries. Several responders from around the world provided details on the incidence of COVID-19–associated mucormycosis (CAM) and shared their clinical perspective on the increased incidence in certain parts of the world. Some responders in India noted that this was likely a result of a misuse of steroids during the treatment of such patients. This theory was also supported by responders from other countries. In Pakistan, several cases of mucormycosis and other fungal infections in patients with COVID-19 were attributed to the combination of viral infection, use of steroids, uncontrolled diabetes, and a long stay in the intensive care unit. In Australia, mucormycosis increases were not documented; however, several cases of another fungal infection were reported in patients with COVID-19. In the United Kingdom, sporadic CAM cases had been reported in patients with COVID-19. EpiCore member reports revealed the incidence of CAM was increasing in geographies across the globe and not limited to the occurrence in India as initially noted by the media [25,26].

## Discussion

### Principal Results

EpiCore provides an innovative mechanism to increase the specificity of signals of events detected across the globe through epidemic intelligence activities from several sources. Over half of all RFIs received a response, and of those, virtually all received responses within 24 hours. Response rates and verification rates increased year by year during the observed period.

### Limitations

Although EpiCore has representation in 161 countries, several areas of the globe are without any responders or have suboptimal numbers. The limitation of responder coverage consequently reduces the number of responses an RFI may receive. To circumvent this situation, RFIs are sent to responders in neighboring geographies when none are in the geography related to the RFI or it has very low numbers of EpiCore members. Currently, RFI generation is limited by the number of requesters, their availability at the time, and their subjective assessment of ongoing multiple global threats. Increasing the number of requestors can help alleviate some of these challenges. Additionally, because most EpiCore responders have human expertise, we recognize that this may hinder the speed with which RFIs about animal and environmental health events are verified. Concerted efforts will continue to recruit responders with animal and environmental health expertise to ensure EpiCore can provide timely and useful responses to RFIs across all One Health sectors. The timely dissemination of information provided via the RFI summary on the public dashboard is a priority for EpiCore. However, the tradeoff between the speed at which the RFI summary is published on the dashboard and the quantity and specificity of information received from responders by leaving the RFI open longer is recognized. To counter this issue, verified RFIs that are made available on the public dashboard can be updated on an ongoing basis with additional information rather than delaying the summary until all details have been received.

### Looking Ahead

EpiCore continues to foster a collaborative approach to support the verification of potential early warning signals and provide information relevant for risk assessment purposes to the growing number of organizations focused on the use of data for epidemic and pandemic intelligence in the future. The ability of all requestors on the EpiCore platform to view all requests and responses regardless of who sends the requests is one way to reduce the duplication of efforts and siloes of information that are common among groups engaged in epidemic and pandemic intelligence.

EpiCore aims to achieve an average time of response to an RFI of less than 24 hours in all regions of the world. Increasing the number of members and their geographic distribution will further enable this goal. Recruitment efforts will continue in the coming years with a particular focus on regions of the world with the fewest EpiCore members. Mechanisms to motivate responder engagement will be considered, including nonfinancial incentives such as increased training opportunities and access to curated web-based trainings and scientific publications. EpiCore will continue to share quarterly digital newsletters to document and disseminate the value of member contributions and provide updates on recently verified events. Additionally, EpiCore will expand its incorporation into human, animal, and environmental training curricula to ensure the next generation of those on the front lines are better positioned to expedite global pandemic and epidemic intelligence.

Certain functionalities within EpiCore may benefit from the application of generative AI tools. The use of machine learning capabilities will be explored and tested against human-curated RFIs to ensure validity and accuracy. Digital disease-detection platforms using machine learning models to identify and aggregate reports of health events through informal sources (eg, social media) [27], can also be leveraged to identify health events for which an RFI should be sent.

### Conclusion

The global demand for data to improve disease prediction, prevention, and control continues to grow. A recent paper authored by the WHO underlines that being better prepared for future pandemics and epidemics will require increased collaboration among stakeholders and investment in collective abilities to detect and understand public health risks [3]. The use of EpiCore by multiple sectors can contribute toward furthering a multi-sectoral approach to epidemics and pandemics (ie, One Health Intelligence). With members spanning human, animal, and environmental health, EpiCore can help the expeditious verification of any health event. EpiCore will continue to provide information for the verification of health events to complement traditional systems and will evolve as needed to better address the global need for situational awareness and risk assessment.

## Abbreviations

CAM: COVID-19–associated mucormycosis
MERS: Middle East respiratory syndrome
MSF: Médecins Sans Frontières
ProMED: Program for Monitoring Emerging Diseases
RFI: request for information
TEPHINET: Training Programs in Epidemiology and Public Health Interventions Network
WHO: World Health Organization

## Data Availability

The data sets generated or analyzed during this study are available from the corresponding author upon reasonable request.
